# Osteoporosis and major fragility fractures (MOF) in sarcoidosis patients: association with disease severity

**DOI:** 10.1007/s40520-023-02589-3

**Published:** 2023-11-04

**Authors:** Carla Caffarelli, Paolo Cameli, Antonella Al Refaie, Caterina Mondillo, Alessandro Versienti, Giuditta Manasse, Elena Bargagli, Stefano Gonnelli

**Affiliations:** 1 Section of Internal Medicine, Department of Medicine, Surgery and Neuroscience, University of Siena, Policlinico Le Scotte, Viale Bracci 2, 53100 Siena, Italy; 2 Respiratory Diseases and Lung Transplantation Unit, Department of Medicine, Surgery and Neuroscience, University of Siena, Siena, Italy

**Keywords:** Major osteoporosis fracture (MOF), Osteoporosis, BMD, Sarcoidosis, Chest X-ray, DLCO

## Abstract

**Background:**

The reports on bone mineral loss or major osteoporosis fracture (MOF) in sarcoidosis are scarce and have conflicting outcomes. This study aimed to evaluate the prevalence and risk factors of MOF in sarcoidosis patients.

**Methods:**

In a single-center cohort of 382 patients with sarcoidosis (55.8 ± 11.6 years) we evaluated bone mineral density at lumbar spine, at femoral neck and at total hip and the presence of MOF. Lung function measurements including diffusion capacity for carbon monoxide (DLCO) were assessed. Chest X-rays were performed and radiological staging was done by Scadding score.

**Results:**

Ninety patients (23.6%) with sarcoidosis have history of a MOF. BMD *T*-scores were lower in sarcoidosis with MOF with respect to those without MOF, but the difference was statistically significant only for BMD at femoral neck (*p* < 0.05). Moreover, BMD values at total hip was positively correlated with DLCO (%) (*p* < 0.001). Prevalence of MOF was higher in patients with sarcoidosis with lung parenchymal involvement (radiological stages 2–4) than in patients with sarcoidosis in chest X-ray stages 0 and 1 (28.3 vs 19.2% respectively, *p* < 0.05). Moreover, multiple regression analyses showed that X-ray Scadding score was positively associated with MOF.

**Conclusions:**

This study shows that MOF represent a common and important complication in patients with moderate/severe sarcoidosis. The chest X-ray evaluation and the pulmonary function test could allow to define the risk of MOF in sarcoidosis patients.

## Introduction

Osteoporosis is defined as a systemic skeletal disease characterized by low bone mass and microarchitectural deterioration of bone tissue, with a consequent increase in bone fragility and susceptibility to fracture [1].

It is associated with an excess of mortality, a decrease in quality of life, and co morbidities; and has a high social and economic burden, representing a major public health problem. Osteoporosis affects mostly postmenopausal women and fewer men. It is mainly related to the normal aging process—primary osteoporosis. Nevertheless, it can also occur due to secondary causes such as bone-affecting treatments, clinical disorders, or lifestyle habits-secondary osteoporosis. Nine million osteoporotic fractures occur worldwide yearly [2]. The last decade there has been a growing awareness of the role of chronic lung diseases as a cause of secondary osteoporosis. In fact, several studies have established that both chronic obstructive pulmonary disease (COPD) and interstitial lung diseases play an important role in the etiopathogenesis of osteoporosis and consequent fragility fracture [3].

Sarcoidosis is a systemic inflammatory disorder characterized by the formation of granulomas, which are clusters of inflammatory cells, typically in the lungs, lymph nodes, and other organs [4].

Several factors contribute to the increased risk of osteoporosis in sarcoidosis patients, including the chronic inflammation associated with the disease and the use of corticosteroids as a treatment. In fact, the chronic inflammation seen in sarcoidosis can disrupt the normal bone remodeling process. Inflammation leads to an imbalance between bone formation and resorption, resulting in increased bone loss and reduced bone quality [5–7]. Cytokines and other inflammatory mediators released during inflammation can directly affect bone cells, leading to decreased bone formation. Moreover, corticosteroids, such as prednisone, are commonly used to suppress the inflammatory response in sarcoidosis. However, long-term use of corticosteroids is associated with a higher risk of osteoporosis and fractures. Corticosteroids can inhibit bone formation, increase bone resorption, and impair the absorption of calcium, resulting in decreased bone density and increased fracture risk [5–7]. Moreover, lung disease can lead to muscle weakness and reduced physical activity due to shortness of breath, fatigue, and overall decreased exercise tolerance. Muscles play a crucial role in maintaining bone strength, and reduced muscle mass and strength can contribute to an increased risk of fractures. However, the data on bone mineral loss or fracture in sarcoidosis have conflicting outcomes. Several studies have reported a high prevalence of fragility fractures and vertebral deformities in patients with sarcoidosis [8–10].

Among MOF in sarcoidosis patients vertebral fractures are the most serious. In fact, vertebral fractures can have various effects on lung diseases depending on the specific circumstances and the severity of the fracture. In particular, the vertebral fractures can cause pain and restrict the movement of the chest wall. This can lead to shallow breathing and decreased lung expansion, resulting in reduced lung function. Moreover, lung diseases such as chronic obstructive pulmonary disease (COPD) or pulmonary fibrosis such as sarcoidosis, can make individuals more susceptible to lung infections [11]. Fractures can further compromise the respiratory system, increasing the risk of complications such as pneumonia, atelectasis, or pleural effusion.

The aim of this single-center cross-sectional study was twofold:

1. To identify the prevalence of major osteoporotic fractures in patients with sarcoidosis.
2. To assess risk factors for major osteoporotic fractures.

## Materials and methods

### Subjects

We studied a cohort of 382 subjects (mean age 55.8 ± 11.6 years) affected by sarcoidosis, referred to the Regional Referral Center for Sarcoidosis and other Interstitial Lung Diseases at Siena University Hospital (Siena, Italy) from January 2018 to December 2022. Diagnosis of sarcoidosis was performed through a multidisciplinary evaluation according to the diagnostic criteria of the ATS/ERS/JRS/ALAT guidelines [12, 13]. All these patients underwent an evaluation of bone status at the outpatient Clinic for Osteoporosis of the Department of Internal Medicine at the University Hospital (Siena, Italy). The patients with a secondary cause of osteoporosis such as hyperthyroidism, hyperparathyroidism, severe chronic renal failure (GFR less than 30 mL/min) and known malignancy were excluded. Other exclusion criteria were history of alcohol abuse and prolonged intake of drugs known to affect bone metabolism such as anticonvulsants, gonadic hormones, anabolic steroids, teriparatide, parathyroid hormone, vitamin D analogs, denosumab or bisphosphonates. Each patient underwent a structured medical interview (including detailed questions on any drugs taken for the treatment of sarcoidosis, smoking history, concomitant diseases, etc.), and pulmonary function evaluation. In addition, height and weight were measured in a standardized manner. Body Mass Index (BMI) was calculated as weight in kilograms divided by the square of height in meters. Written consent was obtained from all participants, and the study was approved by the Institutional Review Board of Siena University Hospital.

### Densitometric measurements

In all subjects we measured BMD at the lumbar spine [LS-BMD] and at femoral subregions (femoral neck [FN-BMD] and total hip [TH-BMD]) using a dual-energy X-ray absorptiometry device (Lunar Prodigy; GE Healthcare, Waukesah, WI, USA). Osteoporosis and osteopenia were diagnosed according to the World Health Organization (WHO) definition: a *T* value lower than − 2.5 was diagnosed as osteoporosis, and a *T* value between − 1.0 but higher than − 2.5 was diagnosed as low bone density. Sex-matched Italian reference data were used for the calculation of *T*-score [14].

### Assessment of vertebral fractures

The information on history of previous fracture was collected during osteoporosis visit. All surveys included details of fracture location, including spine, hip, wrist, clavicle, upper arm/shoulder, rib, pelvis, ankle, upper leg and lower leg. Besides, the occurrence of fractures was assessed by way chart review in the Carestream database, by reviewing progress notes, and by radiological evidence of vertebral fracture. Magnetic Resonance Imaging (MRI), CT with 18F-fluorodeoxyglucose (FDG PET-CT), chest high resolution computed tomography (HRCT) and Computed Tomography (CT) reports were also reviewed. In particular, all radiological images were examined for the presence of any vertebral fracture according to Genant’s method’s [15]. Namely, the vertebrae were identified and morphometry was performed from the fourth thoracic vertebra (T4) to the fourth lumbar vertebra (L4) by marking six points in each vertebral body, corresponding to the four corners and the midpoints of the endplates. The anterior (Ha), mid-vertebral (Hm), and posterior (Hp) heights of each vertebra were measured and the three ratios, Ha/Hp, Hm/Hp, and Hp/Hp-below, were calculated [15]. Two Authors (G-S and C-C) independently evaluated the presence of vertebral fractures. In cases of divergent opinions, consensus was reached by discussion with a radiologist. According to a classification based on the frequency with which they occur, less frequent fractures are considered as minor and to be borne by the skeletal segments with less impact on the overall functional autonomy of the patient (clavicle, upper arm/shoulder, rib, pelvis, ankle, upper leg and lower leg) while they consider themselves to be major osteoporosis fractures (MOF) of the proximal humerus, wrist, proximal femur and spine [16].

### Biochemical parameters

In all subjects blood samples were collected in the morning under fasting conditions to evaluate serum levels of calcium (Ca), phosphate (P), creatinine (Cr), alkaline phosphatase (ALP), 25-hydroxyvitam D (25OHD), Serum1,25-hydroxyvitam2D (1,25OH2D) and intact parathyroid hormone (PTH). At the same time points 24-h urine samples were collected for the evaluation of calcium, phosphate and creatinine. Serum 25OHD was determined by a radioimmunometric method (25-hydroxyvitam D, DiaSorin, MN, USA). In our Institution the intra- and inter-assay coefficients of variation for 25OHD were 6.8 and 9.2%, respectively. Serum1,25(OH)2D was assessed by chemiluminescence immunoassay (LIAISON XL 1,25-Dihydroxyvitamin D, DiaSorin Inc., Stillwater, MN, USA). In our institution the intra- and inter-assay coefficients of variation were 4.1 and 5.3%, respectively. Serum PTH was assessed by an immunoradiometric assay (DiaSorin, Saluggia, Italy). The results were expressed in picograms per millilitre, and the intra and inter-assay coefficients of variation were 3.6 and 4.9%, respectively. Urinary calcium, phosphate and creatinine were determined by a colorimetric method (Cobas C311 analyser, Roche Diagnostics, USA) in 24-h urine samples.

### Lung function assessment

All participants underwent pulmonary function tests performed according to the American Thoracic Society/European Respiratory Society (ATS/ERS) standards [17, 18], using a Jaeger body plethysmograph with corrections for temperature and barometric pressure. Forced vital capacity (FVC), forced expiratory volume in 1 s (FEV1), FEV1/FVC and lung diffusion capacity for carbon monoxide (DLCO) were assessed. All parameters were expressed as percentages of predicted values.

All subjects with sarcoidosis performed a chest X-ray with radiological staging according to Scadding criteria [19]. The radiological classification was linked to sample detection in a standard manner according to widely accepted criteria: stage 0, normal; stage 1, bilateral hilar adenopathy without parenchymal involvement; stage 2, bilateral adenopathy and parenchymal infiltration; stage 3, parenchymal infiltration; and stage 4, pulmonary fibrosis associated with sarcoidosis [19]. The stage at presentation is generally considered to correlate with prognosis, in particular Stage I and II generally have a very good prognosis (they often regress spontaneously or are very responsive to treatment) whereas stage III and especially stage IV are less successful [19]. Clinical phenotyping was performed according to Clinical Outcome States (COS) classification [20], on the basis of the disease persistence and the need for systemic therapy. We applied the classification of Schupp et al. [21], dividing patients into 5 phenotypes according to disease localization: group 1, abdominal; group 2, ocular, central nervous system, cardiac, or cutaneous; group 3, musculoskeletal and cutaneous; group 4, hilar lymph adenopathy and intrathoracic; and group 5, extrathoracic disease. These five new clinical phenotypes will be useful to recruit homogenous cohorts in biomedical studies and above all for better management of the disease [21].

### Statistical analysis

The Kolmogorov–Smirnov test was used to verify the normality of the distribution of the outcome variables. All the variables were normally distributed and were expressed as mean ± SD. The significance between the means was tested using Student’s *t*-test. Categorical variables were compared by Chi-square test or Fisher’s exact test, as appropriate. The correlations between the groups were analyzed with the Pearson’s correlation test. Multiple linear regression models were used to assess the association of independent predictors such as sex, BMI, creatinine, vitamin D, FVC (%), FEV1 (%), DLCO (%), COS classification, Radiological stages, therapy for sarcoidosis, prednisone dosage to the presence of osteoporosis and MOF. All tests were two-sided, and *p* < 0.05 was considered statistically significant. All statistical tests were performed using SPSS 10.1 statistical software (SPSS 10.1).

## Results

The clinical and biochemical characteristics of sarcoidosis patients with or without MOF are shown in Table 1. There were no significant differences between the two groups for BMI, calcium, phosphate and vitamin D serum levels. Sarcoidosis patients with MOF had serum levels of creatinine, 1,25OHVitD and serum PTH slightly higher with respect to those without MOF (*p* < 0.05). As expected patients with sarcoidosis and MOF were older than those without MOF.

**Table 1:** Clinical and biochemical characteristics of the patients with sarcoidosis with or without major osteoporotic fracture (MOF)

	All (*N* = 382)	Without MOF (*N* = 292)	With MOF (*N* = 90)
Sex (F/M)	229/153	178/114	51/39
Age (years)	55.8 ± 11.6	54.3 ± 11.3	60.7 ± 11.4**
BMI (Kg/m^2^)	26.4 ± 4.6	26.3 ± 4.6	26.7 ± 4.4
Calcium (mg/dL)	9.45 ± 0.71	9.47 ± 0.77	9.41 ± 0.48
Phosphate (mg/dL)	3.44 ± 0.50	3.45 ± 0.48	3.41 ± 0.56
Creatinine (mg/dL)	0.99 ± 0.91	0.93 ± 0.57	1.18 ± 1.55*
Urine calcium (mg/24 h)	199.58 ± 123.5	190.59 ± 113.56	202.56 ± 126.80
Urine phosphate (mg/24 h)	741.42 ± 352.79	637.30 ± 330.29	741.42 ± 352.79
Urine creatinine (mg/24 h)	1124.48 ± 643.17	1061.61 ± 568.93	1149.12 ± 670.22
Alalkaline phosphatase (U/L)	82.69 ± 44.20	81.85 ± 45.85	85.62 ± 39.69
25OHD (ng/mL)	25.88 ± 13.88	25.39 ± 13.18	27.14 ± 15.72
1,25(OH)D2 (pg/mL)	42.35 ± 16.83	38.54 ± 15.77	56.33 ± 11.03*
PTH (pg/mL)	36.25 ± 13.64	34.57 ± 13.14	42.12 ± 14.62*

**Fig. 1:**
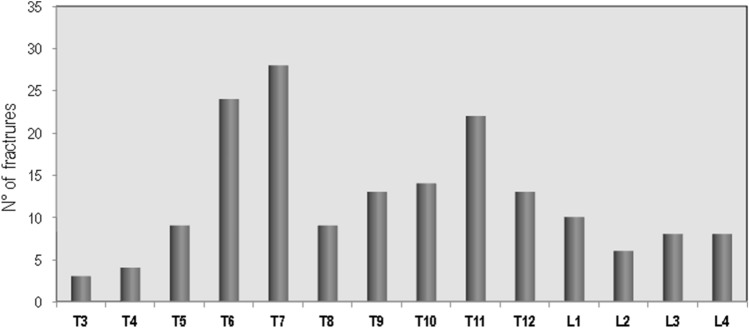
Distribution of vertebral fractures in sarcoidosis patients with MOF

Ninety patients (23.6%) with sarcoidosis have presented a major osteoporosis fracture. In particular, the MOF reported involved vertebrae (*N* = 81), femur (*N* = 4), wrist (*N* = 7) and humerus (*N* = 5). Moreover, 52 patients had history of more than one MOF. The prevalence of vertebral fractures in patients with sarcoidosis, by spinal location, is shown in Fig. 1. The distribution of vertebral fractures along the spine showed a peak prevalence at T6 and T7; and another peak prevalence was found at T11.

**Fig. 2:**
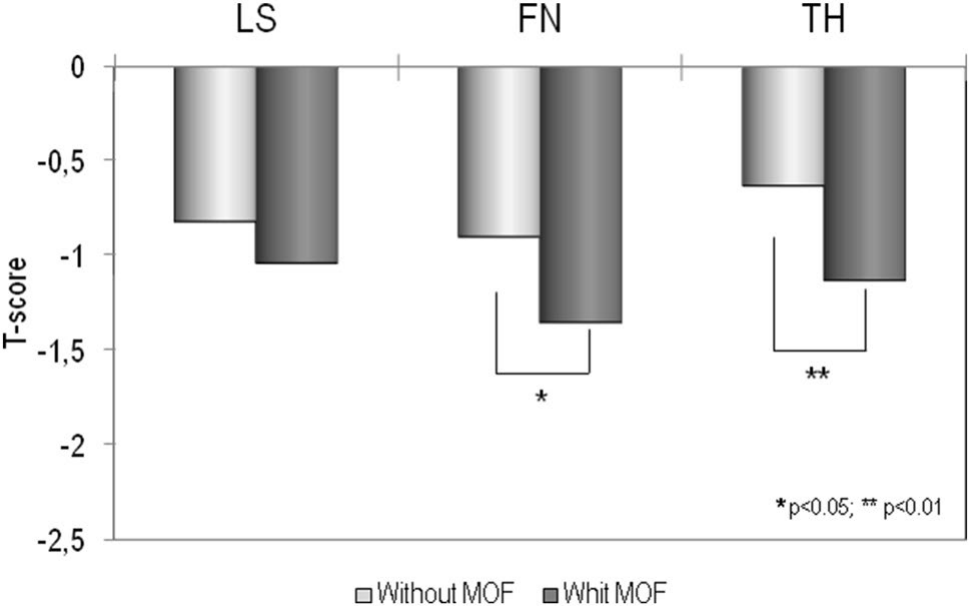
Values of BMD expressed as *T*-score at lumbar spine (LS), at femoral neck (FN) and at total hip (TH) in patients affected by sarcoidosis with or without major osteoporotic fracture (MOF)

Figure 2 shows the mean values of BMD at different skeletal sites, expressed as *T*-score, obtained in patients affected by sarcoidosis with or without MOF. It is evident that BMD *T*-scores were lower in patients affected by sarcoidosis with MOF respect to those without MOF, but the difference was statistically significant only for BMD-FN (*p* < 0.05) and BMD-TH (*p* < 0.01).

**Fig. 3:**
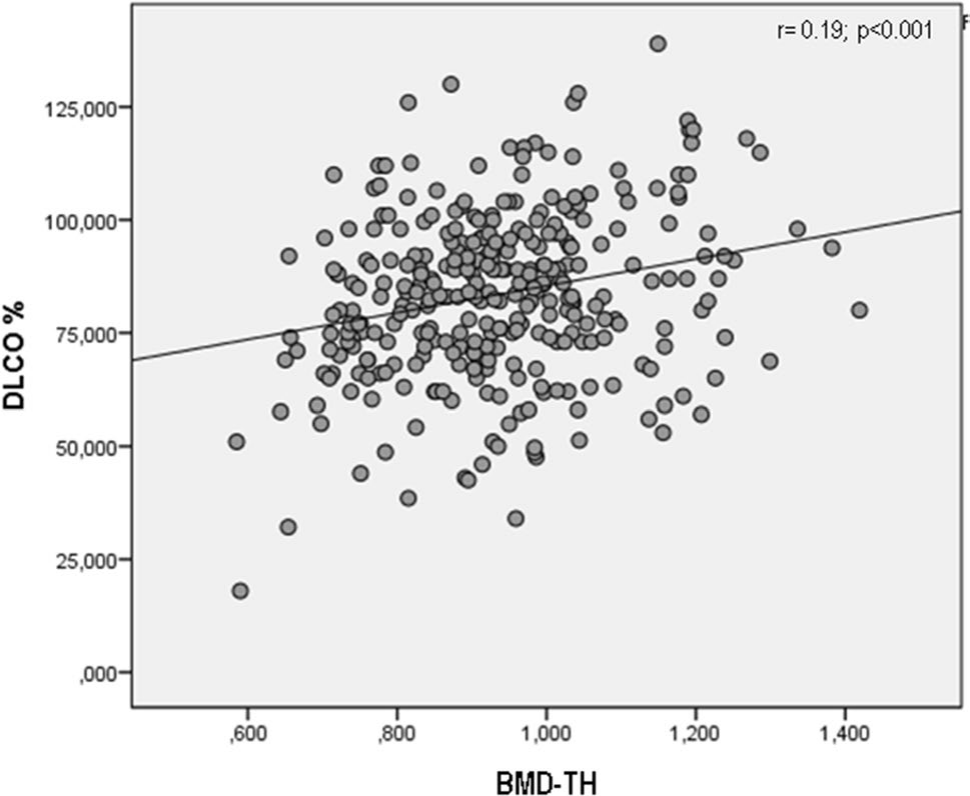
Age and BMI adjusted partial correlation of DLCO% and BMD-TH values in subjects with sarcoidosis

Figure 3 shows the significant positive age and BMI adjusted partial correlations of BMD-TH values with DLCO (%) (*p* < 0.001). Also BMD-LS and BMD-FN were significantly correlated with DLCO% (*r* = 0.20 *p* < 0.001 and *r* = 0.18 *p* < 0.001 respectively). Instead, no significant associations between BMD values with FVC (%) and FEV1 (%) were observed.

**Fig. 4:**
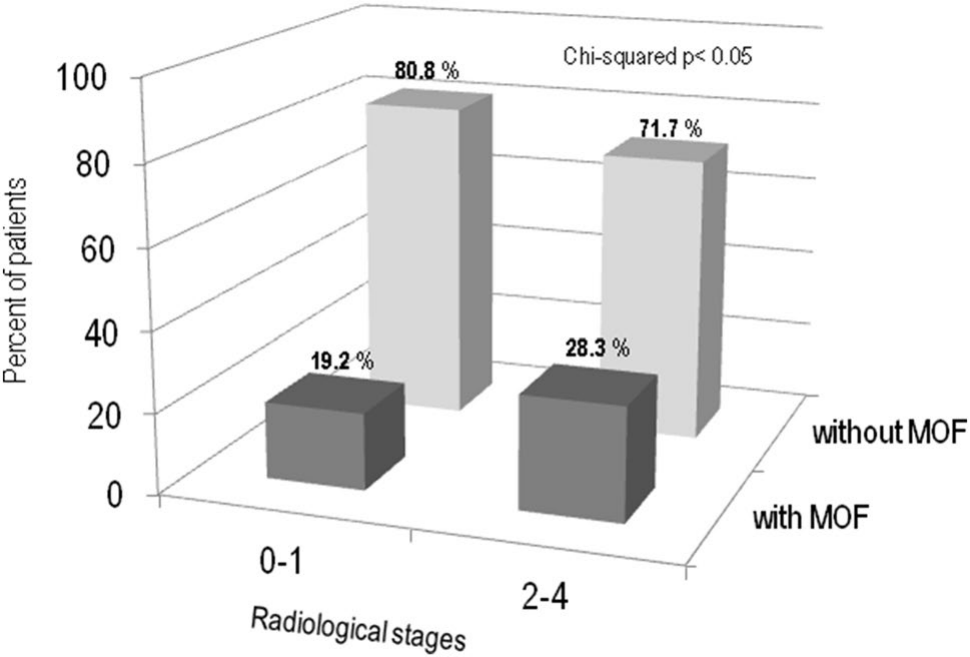
Percentage of sarcoidosis patients with or without MOF on the basis of according to the chest radiograph type according to Scadding score

Figure 4 shows the percentage of sarcoidosis patients with or without MOF on the basis of the chest radiograph type according to Scadding score. As suggested by Sweiss et al. [21], patients in chest X-ray stages 0 and 1 were grouped together, as were patients in stages 2, 3 and 4; the latter shared parenchymal involvement. It’s evident that the prevalence of MOF was higher in patients with sarcoidosis with lung parenchymal involvement (radiological stages 2–4) than in patients with sarcoidosis in chest X-ray stages 0 and 1 (28.3 vs 19.2% respectively, *p* < 0.05).

In multiple regression analysis, statistically significant associations were found for predictors of osteoporosis (model 1) and of MOF (model 2). In particular, multiple regression analyses showed that BMI was positively associated with osteoporosis, while DLCO (%) was negatively associated (Table 2). Regarding predictor of MOF, multiple regression analyses showed that X-ray Scadding score was positively associated with MOF, while BMD-TH and therapy use was negatively associated (Table 2).

**Table 2:** Multiple linear regression analysis of predictors for osteoporosis and major osteoporotic fracture (MOF) in 382 patients affected of sarcoidosis

Variable	Undestandardized coefficient, *b*	95% CI	*p*
*Osteoporosis*			
BMI	0.024	0.002 to 0.046	0.032
DLCO %	− 0.212	− 0.416 to − 0.009	0.041
*Major osteoporotic fracture (MOF)*			
BMD-TH	− 0.092	− 0.148 to − 0.036	0.002
X-ray Scadding score	0.046	0.005 to 0.088	0.030

Whole set of variables included into the osteoporosis model: sex, BMI, creatinine, vitamin D, FVC (%), FEV1 (%), DLCO (%), COS classification, radiological stages, therapy for sarcoidosis, prednisone dosage. Whole set of variables included into the MOF model: sex, BMI, creatinine, vitamin D, BMD-LS, BMD-FN, BMD-TH, FVC (%), FEV1 (%), DLCO (%), COS classification, radiological stages, therapy for sarcoidosis, prednisone dosage

## Discussion

Osteoporosis and the occurrence of fragility fracture are major co-morbidities and extra-pulmonary manifestations concern in patients with pulmonary disease [23].

To the best of our knowledge, this is the first study that evaluated the prevalence and the clinical impact of MOF in a large cohort of sarcoidosis patients. In particular, our data show that about a quarter of sarcoidosis patients (23.56%) had a MOF. According to a classification based on the frequency with which they occur, MOF consider a grouping of the most common fractures which include hip, clinical vertebral, distal forearm and proximal humerus fractures [16, 24]. Moreover, recent MOF is an important predictor of “imminent fracture risk,” which has been defined as high risk of near-term fracture within the next 12 to 24 month [25]. Prior fracture type impact risk of subsequent fracture that is largely independent of bone mineral density; moreover, site of re-fracture is similar to original major osteoporotic fracture [25]. In particular, the risk of MOF after a first MOF is increased over the whole follow-up, but the imminent risk is even higher [26].

An important finding of this study is that approximately a quarter of sarcoidosis patients had a MOF and over a fifth of them had at least one vertebral fracture. Similarly, in the Maastricht study at baseline 20% of patients with sarcoidosis had vertebral fractures while non-vertebral fractures were rare [27]. Moreover, a 4-year follow-up study carried out in the some population reported that the prevalence of vertebral fractures increased from 20 to 32%, while non-vertebral fractures remained infrequent [28]. Also a cross-sectional French study on consecutive 142 sarcoidosis patients reported that fragility fractures occurred in 23.5% of patients despite a normal BMD [8]. Moreover, the study by Bours et al., carried out using a database of general practitioners across the United Kingdom, reported that the risk of any fractures was similar in patients with sarcoidosis and in matched controls while the risk of vertebral fractures was significantly increased and the risk of non-vertebral fractures marginally reduced [10]. In our study population, vertebral fractures in patients with sarcoidosis predominantly affect the thoracic spine (with peaks at the level of T6, T7 and T11) and, therefore, with a distribution similar to that reported in previous studies carried out in patients with severe pulmonary interstitial diseases or awaiting lung transplant [29]. The prevalent distribution of vertebral fractures at the thoracic level may negatively influence the course of the disease because these fractures, especially those of moderate/severe degree, have deleterious effects on pulmonary performance by reducing lung volume so contributing to a restrictive ventilatory defect [30–32]. Moreover, individuals who experience a vertebral fracture are at risk for future vertebral fracture and the risk is increased with the number and severity of prevalent vertebral fractures [25]. A recent review has reported that severe clinical and personal consequences may arise in the aftermath of VFs, including back pain, physical functional limitations and a poorer physical health-related QoL [33]. Although the pathophysiological mechanisms responsible for increased bone fragility in sarcoidosis has not yet been clarified. BMD is considered a major determinant of bone strength. Indeed literature data on the relationship between sarcoidosis and BMD are conflicting; in fact, even if most of the studies have found reduced BMD values [5–7, 34], others have not observed differences compared to a control population [8, 27, 28]. However, recent studies have shown that in sarcoidosis patients BMD values are influenced by the severity of the disease. In fact, a recent paper by Malaise O et al. have evaluated the functional indices associated with emphysema on pulmonary function tests can identify COPD patients with osteoporosis. This study reported that lung function impairment determined by associating hyperinflation with impaired diffusion capacity and transfer coefficient (DLCO < 70%, DLCO/AV < 80% and TLC > 115%), is an independent risk factor for hip osteoporosis. [35]

The severity of sarcoidosis has been suggested as a potential risk factor for the development of osteoporosis and major fragility fractures (MOF). In fact, we have found that patients with more severe sarcoidosis, as indicated by lung involvement, multiorgan involvement, or higher disease activity, may be at a higher risk of developing osteoporosis and experiencing MOF. However, it is important to note that the association between sarcoidosis severity and osteoporosis/MOF is not fully understood and requires further investigation. Other factors, such as age, gender, menopausal status (in women), vitamin D deficiency, and physical inactivity, can also contribute to the development of osteoporosis in sarcoidosis patients [34].

Another important finding in this study is represented by the close correlation between the worsening of the respiratory decalages, expressed by the DLCO%, and the reduction of femoral BMD; moreover, the multiple linear regression model confirms that the DLCO% is able to predict the risk of osteoporosis. Multiple regression analysis also reported that BMI shows a protective effect against osteoporosis. This finding seems to be in agreement with a previous study by Nuti et al. witch reported that an association between the reduction in BMI and the risk of vertebral fracture in COPD patients [3]. Moreover, Compston et al. in the Global Longitudinal study of Osteoporosis in Women (GLOW) study reported that high BMI led to a decrease in the relative risk for fragility fracture at axial sites [36].

Moreover, the extent of chest radiology involvement evaluated according by Scadding’s criteria is able to predict the risk of MOF in sarcoidosis patients independently of BMD. The better association of femoral BMD with disease severity and MOF with respect to lumbar BMD may be explained by the increased occurrence of spondyloarthritis in sarcoidosis patients [37].

Our study has some limitations. Firstly, the cross-sectional nature of the study does not allow the establishing of any causality relationships between the parameters. Secondly, the lack of data about markers of inflammation. Nevertheless, this study presents several strengths: firstly, the large study sample of a rare condition such as sarcoidosis disease; secondly, it was a single-centre study and this may ensure its internal validity; thirdly, has confirmed that the severity of pulmonary involvement is related to the risk of osteoporosis and fragility vertebral fractures.

Our data obtained from a large cohort of sarcoidosis patients confirm that MOF and particularly vertebral fracture represent a common and important complication in patients with moderate/severe sarcoidosis. In these patients, the radiological evaluation according to the Scadding’s criteria and the measurements of DLCO could allow to define the risk of MOF and to implement adequate therapeutics strategies.

## Data Availability

The data that support the findings of this study are available from the corresponding author upon reasonable request.
